# Hampton’s Hump: Hypoxia with Lung Consolidation Mimicking Pneumonia

**DOI:** 10.31662/jmaj.2021-0079

**Published:** 2021-12-03

**Authors:** Michiko Campbell Kawasaki, Junki Mizumoto

**Affiliations:** 1 Department of Family Practice, Ehime Seikyo Hospital, Matsuyama, Japan; 2 Department of Medical Education Studies, International Research Center for Medical Education, Graduate School of Medicine, The University of Tokyo, Tokyo, Japan

**Keywords:** Diagnostic Reasoning, Emergency Medicine, Hampton’s Hump, Pulmonary Embolism, Respiratory Disease

A 72-year-old man presented with mild dyspnea since 1 day prior. He did not experience chest pain, fever, or cough. His vital signs were remarkable in tachycardia, tachypnea, and hypoxia. Chest X-ray revealed slight consolidation at the right lower lung. (Figure 1A) Contrast computed tomography revealed an isolated patchy consolidation at the right lower lobe adjacent with the pleura (Figure 1B and 1C) and multiple defects within the bilateral pulmonary arteries. (Figure 2) The patients was diagnosed with pulmonary embolism (PE). Anticoagulant therapy was administered, and the patient’s conditions improved.

**Figure 1.**
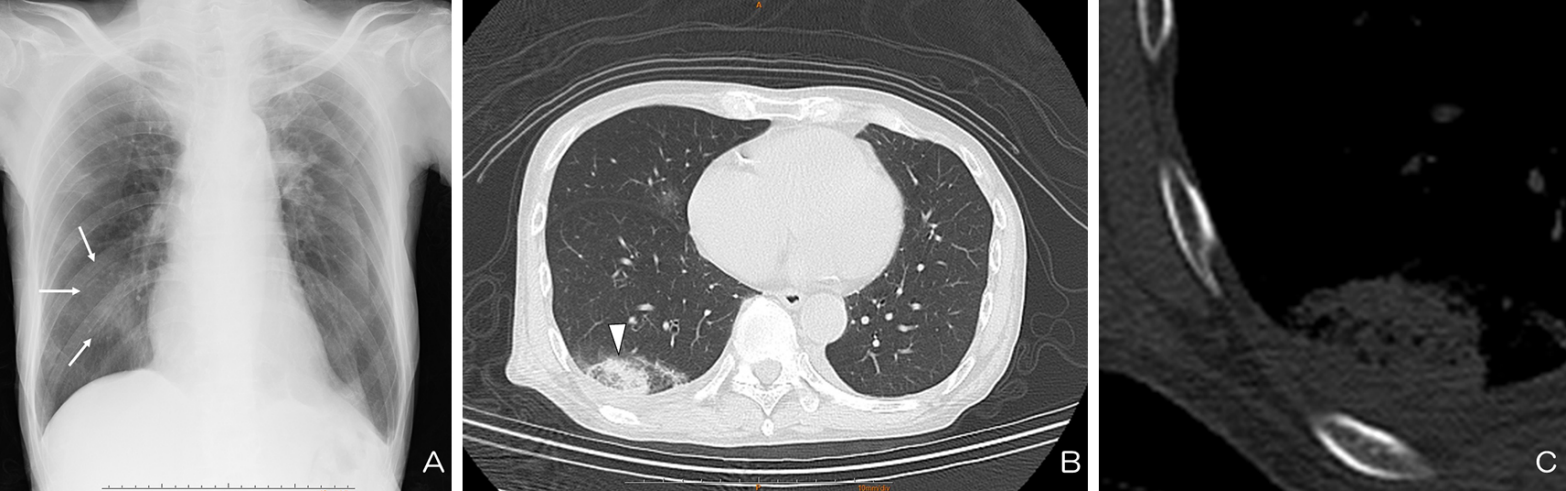
(A) Slight consolidation in chest X-ray (arrow) (B) Isolated patchy consolidation adjacent with the pleura in computed tomography (arrowhead) (C) A wedge-shaped consolidation without the expected apex seen well in the mediastinal. A possible reason of sparing the apex is collateral supply from the bronchial arterial circulation.

**Figure 2.**
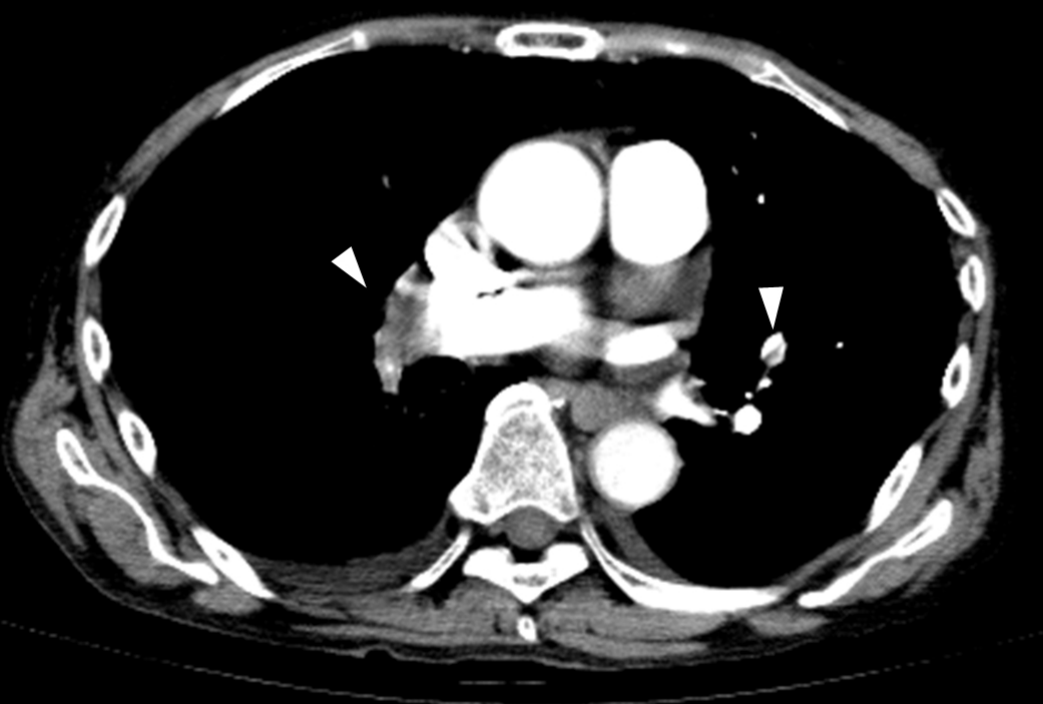
Multiple defects within the bilateral pulmonary arteries (arrowhead).

PE sometimes presents with isolated opacity along the pleural surface, called Hampton’s hump ^(1)^. It represents small pulmonary infarction. However, it is less frequently seen due to collateral circulation in the lung parenchyma. This sign strongly suggests the presence of PE, although it has low sensitivity ^(2)^. Physicians should note that not all consolidations in patients with hypoxemia are due to inflammatory or interstitial disease.

## Article Information

### Conflicts of Interest

None

### Author Contributions

Kawasaki CM and Mizumoto J wrote the manuscript.

### Informed Consent

Informed written consent was obtained from the patient for publication of this report and any accompanying images.
